# Blood mercury levels and fish consumption in pregnancy: Risks and benefits for birth outcomes in a prospective observational birth cohort

**DOI:** 10.1016/j.ijheh.2016.05.004

**Published:** 2016-08

**Authors:** Caroline M Taylor, Jean Golding, Alan M Emond

**Affiliations:** Centre for Child and Adolescent Health, School of Social and Community Medicine, University of Bristol, Bristol, UK

**Keywords:** ALSPAC, Avon Longitudinal Study of Parents and Children, B-Hg, blood mercury, B-Se, blood selenium, LBW, low birth weight, SGA, small for gestational age, ALSPAC, Pregnancy, Mercury, Birth outcomes, Fish consumption, Guidelines

## Abstract

**Background:**

To avoid exposure to mercury, government advice on fish consumption during pregnancy includes information on fish species to avoid and to limit, while encouraging consumption of least two portions of fish per week. Some women may, however, chose to avoid fish completely during pregnancy despite potential benefits to the fetus.

**Objectives:**

Our aims were to evaluate the effects of blood mercury levels in pregnant women on birth outcomes in the UK, and to compare outcomes in those who ate fish with those who did not.

**Methods:**

Pregnant women were enrolled in the Avon Longitudinal Study of Parents and Children (ALSPAC). Whole blood samples for singleton pregnancies with a live birth were analysed for Hg by inductively coupled plasma dynamic reaction cell mass spectrometry (n = 4044). Fish intake was determined by a food frequency questionnaire during pregnancy. Data collected on the infants included anthropometric variables and gestational age at delivery. Regression models were adjusted for covariates using SPSS v23.

**Results:**

There were no significant associations of maternal blood Hg level with birthweight, head circumference or crown–heel length in adjusted linear regression models. Similarly, there were no increased odds of low birthweight or preterm delivery in adjusted logistic regression models. When the models were repeated after stratification into fish-eaters and there were no associations except for a negative association with birthweight in non-fish-eaters (unstandardised B coefficient −58.4 (95% confidence interval −113.8, −3.0) g, p = 0.039).

**Conclusion:**

Moderate mercury levels in pregnancy were not associated with anthropometric variables, or on the odds of low birthweight or preterm birth. Fish consumption may have a protective effect on birthweight. Consumption of fish in line with government guidelines during pregnancy should be encouraged.

## 1 Introduction

Mercury is a cumulative neurotoxin that is present in the environment through a variety of natural and anthropogenic sources: mercury-containing aerosols are released from volcanic activity and from the weathering of rocks, while human activities such as mining and manufacturing processes also contribute to levels in the environment (Hylander and Meili, 2003, Nriagu and Becker, 2003). Additional exposure can occur through elemental mercury vapour from dental amalgams and in some cases from the use of traditional herbal medicines and skin creams (Copan et al., 2015, Golding et al., 2013). Further exposure to mercury at a population level occurs through diet, particularly from fish that are long-lived and high in the food chain (Castano et al., 2015, Golding et al., 2013). High level exposure to mercury can result in significant neurological and behavioural disorders, including tremors, memory loss, neuromuscular changes, renal and thyroid disorders, and death; effects at more moderate levels of exposure are likely to be more subtle, and may include adverse effects on neurological function, the cardiovascular system and immune function (Karagas et al., 2012).

Mercury readily crosses the placenta and fetal levels have been found to be greater than maternal levels (Ding et al., 2013). Evidence of acute effects on the developing fetus were seen following the Minamata disaster in Japan in the 1950s, in which mercury-containing by-products of a manufacturing process were released into the local bay: pregnant women who consumed contaminated seafood had a high prevalence of fetal neurotoxicity and abnormalities such as microcephaly, blindness, mental and physical disabilities (Harada, 1964). In other observational studies, detrimental effects on varied aspects of neurocognition over a range of more moderate prenatal exposure levels have been described (Jedrychowski et al., 2007, Lederman et al., 2008, Oken et al., 2008), although these findings have not always been consistent (Golding et al., 2016, Plusquellec et al., 2010).

While it is recognised that fish-eating is associated with higher levels of mercury exposure (Gundacker et al., 2010, Soon et al., 2014), fish is also an important source of several beneficial nutrients, including long-chain polyunsaturated fatty acids (particularly docosahexaenoic acid and arachidonic acid), choline, iodine, selenium and vitamin D, which are all critically important during pregnancy (Clarkson and Strain, 2003). Fish is also a major source of protein for some populations. This has resulted in a difficult task for government bodies in developing recommendations on eating fish during pregnancy, in which the beneficial effects of fish must be balanced against the potentially detrimental effects from the mercury content of different species of fish at different locations. This has resulted in somewhat confusing national advice (for example, NHS Choices (2015); US Food and Drug Administration, 2004, US Food and Drug Administration, 2014), which may in turn result in pregnant women reducing their fish intake or avoiding it completely (Oken et al., 2003, Shimshack and Ward, 2010).

There are relatively few studies on the effect of moderate or low levels of maternal blood or cord blood/tissue mercury levels on birth outcomes such as birth weight, birth length, head circumference or gestational age (Karagas et al., 2012) (Supplementary Table A1). Such studies have generally found no associations of maternal blood mercury with these outcomes (Al-Saleh et al., 2014b; Ding et al., 2013; Gundacker et al., 2010, Lederman et al., 2008, Lucas et al., 2004, Wells et al., 2016), with a few exceptions, mainly negative associations with birthweight (Lee et al., 2010, Ramon et al., 2009). Some studies have also included data on maternal fish consumption in addition to maternal or cord blood mercury levels during pregnancy (Al-Saleh et al., 2014a, Ding et al., 2013; Gundacker et al., 2010, Lederman et al., 2008, Lee et al., 2010, Ramon et al., 2009), but few of these have stratified or adjusted for fish consumption (Al-Saleh et al., 2014a, Lederman et al., 2008, Ramon et al., 2009). These studies adjusting for fish consumption have generally shown no associations of birth outcomes with prenatal mercury exposure (Supplementary Table A1).

It has been suggested that selenium, of which fish is a rich source, may have a protective effect against the toxic effects of mercury by sequestering mercury, thus reducing its biological availability and preventing the inhibition of selenium-dependent enzymes such as glutathione peroxidase (Ralston and Raymond, 2010). This has been not been studied extensively in relation to birth outcomes, but there was little evidence for mercury–selenium interactions in association with birth outcomes in a cohort of women with moderate mercury levels (Al-Saleh et al., 2014a). This requires further investigation.

The aims of this study were to examine the associations of moderate prenatal mercury exposure in a UK population of pregnant women on birth outcomes (head circumference, crown–heel length, birth weight, low birth weight, preterm birth), and to further study associations with moderate fish-eating and blood selenium levels. An additional aim was to study associations with oily-fish-eating.

## 2 Methods

We first modelled associations of exposure to mercury during pregnancy using maternal blood levels during pregnancy for continuous variables (head circumference, crown–heel length, birth weight) in the newborn infant with adjusted linear regression analyses. Dichotomous variables (low birth weight and preterm delivery) were modelled with adjusted logistic regression analysis. Model 1 included adjustment for covariates such as maternal age, educational attainment, smoking and parity. Model 2 included additional adjustment for maternal blood selenium. Models 1 and 2 were repeated with stratification for fish-consumption versus non-consumption, and for oily-fish-consumption vs non-consumption.

### 2.1 The ALSPAC study

The sample was derived from the ALSPAC study, a population-based study investigating environmental and genetic influences on the health, behaviour and development of children. This database provided an opportunity to include a greater number of participants than has been reported on before and includes a wide range of social and demographic information to enable the most appropriate selection of covariates. All pregnant women in the former Avon Health Authority with an expected delivery date between 1 April 1991 and 31 December 1992 were eligible for the study; 14,541 pregnant women were enrolled, resulting in a cohort of 14,062 live births (Boyd et al., 2013). The social and demographic characteristics of this cohort were similar to those found in UK national census surveys (Fraser et al., 2013). Further details of ALSPAC are available at www.bris.ac.uk/alspac and the study website contains details of all the data that are available through a fully searchable data dictionary (http://www.bris.ac.uk/alspac/researchers/data-access/data-dictionary). Ethics approval for the study was obtained from the ALSPAC Ethics and Law Committee and the Local Research Ethics Committees.

### 2.2 Collection, storage and analysis of blood samples

Whole blood samples were collected in acid-washed vacutainers (Becton and Dickinson, Oxford, UK) by midwives as early as possible in pregnancy. Whole blood samples were stored in the original tube at 4° C at the collection site before being transferred to the central Bristol laboratory within 1–4 days. Samples were at ambient temperature during transfer (up to 3 h). They were then stored at 4° C until analysis. Details of the analysis have been reported (Taylor et al., 2013). In brief, inductively-coupled plasma mass spectrometry in standard mode (R. Jones, Centers for Disease Control and Prevention (CDC), Bethesda, MD, USA CDC; Method 3009.1) was used to measure blood levels of Hg and Se with appropriate quality controls. The analyses were completed for 4134 women for Hg and 4287 for Se. One sample had an Hg level below the limit of detection: this was assigned a value of 0.7 times the lower limit of detection (LOD/√2) (Centers for Disease Control and Prevention, 2005; Hornung and Reed, 1990). No samples were below the limit of detection for Se.

### 2.3 Pregnancy outcomes

Newborn head circumference and crown–heel length were measured by trained study staff where the mother gave permission or if these data were missing, the values were extracted from the medical records by trained study staff. Birth weight was derived from obstetric data and from central birth notification data: where values disagreed by <100 g then the lowest value was accepted; if the values disagreed by >100 g then the value was coded as missing. Study staff were blinded to the maternal blood mercury (B-Hg) and blood selenium (B-Se). Length of gestation was based on last menstrual period date, ultrasound assessment or other clinical indicators. Where there was conflict of ≥14 days between the maternal report and ultrasound assessment, an experienced obstetrician reviewed the clinical records and made a best estimate. Low birth weight (LBW) was defined as birth weight <2500 g and preterm birth as <37 weeks gestation.

### 2.4 Questionnaires

The mothers received four postal self-completion questionnaires during pregnancy. The questionnaires are available from the study website (http://www.bristol.ac.uk/alspac/researchers/questionnaires/). A questionnaire sent to the mother at 32 weeks’ gestation included a food frequency questionnaire (FFQ) (Rogers and Emmett, 1998) comprising 103 food and drink items including three items related to seafood: white fish, oily fish and shellfish. The participants were given guidelines to classify the three types using seafoods that were most prevalent in the UK. Thus oily fish was described as including ‘salmon, mackerel, sardines, trout, herring, pilchards, trout, tuna, etc’; white fish as including ‘cod, haddock, plaice, fish fingers, etc.’ and shellfish as including ‘prawns, mussels, cockles, crab, etc.’. The woman was asked how frequently she was currently eating each of these types of seafood, with options: ‘Not at all; About once in 2 weeks; 1–3 times a week; 4–7 times a week; More than once a day’. ‘Non-fish-eaters’ were categorised as those who responded ‘Never/Rarely’ to the FFQ for both white fish and oily fish consumption; ‘Fish-eaters’ were those who responded ‘Once in 2 weeks/1–3 times per week/4–7 times per week/More than once per day’ for white fish and/or oily fish consumption. ‘Non-oily-fish-eaters’ were categorised as those who responded ‘Never/Rarely’ for oily fish consumption; ‘Oily-fish-eaters’ were categorised as those who responded ‘Once in 2 weeks/1–3 times per week/4–7 times per week/More than once per day’ for oily fish consumption.

Information collected on environmental and lifestyle factors from questionnaires during pregnancy included data on age, parity, social class, highest educational qualification, alcohol consumption, cigarette smoking, etc. Data on cigarette smoking were taken from a questionnaire completed at 18 weeks’ gestation in response to a question on the number of cigarettes smoked in the previous 2 weeks (16–18 weeks’ gestation).

### 2.5 Statistical analysis

Participants were excluded from the study if they did not have a singleton live birth (Supplementary Figure A1). Statistical analysis was done with SPSS version 23 (IBM Corp., Chicago, IL, USA). Values are reported as mean ± SD. Chi-square tests were used to analyse differences in categorical data, and two-sided *t*-tests and were used to compare continuous values.

The distribution of maternal B-Hg approximated to normal (Golding et al., 2013). Univariate and multivariate linear regression models were used to examine the relationship of B-Hg on head circumference, crown–heel length and birth weight. Logistic regression analysis was used to examine the effect of the binary variables preterm (<37 weeks gestation), LBW (<2500 g), and LBW in term deliveries by tertiles of B-Hg. Model 1 included the following confounders: maternal age, maternal height, maternal body mass index, maternal educational attainment, parity (0 vs ≥1), gestational age at delivery (weeks), being a smoker (yes/no), alcohol consumption (number of units per week) and sex of the baby. These factors were chosen as being consistently associated with birth weight, length of gestation, fetal growth retardation and other measures including low birth weight, preterm delivery and small for gestational age (SGA) at birth as well as B-Hg. We did not include maternal ethnicity in any of the models because of the low numbers of ethnic minority participants included in the sample (non-white 2.4%). Model 2 also included maternal B-Se. Since there was collinearity between fish consumption and B-Hg, models 1 and 2 were repeated after stratification for: (1) those who ate oily and/or white fish (fish-eaters)/those who ate no fish (non-fish-eaters); and (2) those who ate oily fish (oily-fish-eaters)/those who ate no oily fish (non-oily-fish-eaters).

Regression diagnostics were used to check that the models fitted the observed data well and to identify any cases that had undue influence on the model.

## 3 Results

### 3.1 Characteristics of mothers and offspring

There were 4044 singleton live births included in the study. Mothers with data on B-Hg were broadly similar to those without, except that they had a higher educational attainment and were slight older (Golding et al., 2016). For mothers with a B-Hg measurement, there were no differences in characteristics between those included and those not included in the study (Supplementary Table A2). As reported previously, the mean B-Hg level was similar to those reported in other developed countries (Taylor et al., 2014).

Maternal B-Hg increased with increasing consumption of white fish and of oily fish (Table 1) with a marked increase for non-consumers to those who ate fish once every 2 weeks and with little difference between those who ate fish 1–3 times and >4 times per week. The characteristics of the mothers and offspring included in the study, and when stratified into maternal fish-eaters and non-fish-eaters, are shown in Supplementary Table A3. Mothers who ate fish during pregnancy had a significantly higher B-Hg level (+32.9%) than those who did not (Supplementary Table A3). The offspring of fish-eaters had a greater mean birth weight (+87.6 g, p < 0.001), head circumference (+0.2 cm, p = 0.020) and crown–heel length (+0.4 cm, p < 0.001) than those of non-fish-eaters, but there were no differences in mean gestational age and prevalences of preterm birth or LBW (in all deliveries and in term deliveries only) (Supplementary Table A3). The mean B-Hg of those who had a preterm birth was lower than for those who did not, but the significance level was weak (1.94 ± 1.03 vs 2.09 ± 1.09 μg/l, p = 0.059); the mean B-Hg of those who delivered a LBW baby was not different from those who did not (1.96 ± 0.93 vs 2.09 ± 1.10 μg/l, respectively, p = 0.133) or from those with a LBW baby that was not preterm (1.91 ± 0.71 vs 2.09 ± 0.10, respectively p = 0.194). The mean B-Hg of the 18 mothers who did not have a live birth and were excluded from the study was not different from those that did have a live birth (2.11 ± 1.29 vs 2.07 ± 1.10, respectively, p = 0.881).

### 3.2 Regression models for B-Hg and birth outcomes

In univariate linear regression models, there were significant positive associations between maternal B-Hg and birth weight, head circumference and crown–heel length (Table 2). However, these association were all attenuated after adjustment (model 1; Table 2). Similarly, in univariate logistic regression models, there was a significant trend for a protective effect of B-Hg tertile on the odds of a preterm birth, but this was attenuated with adjustment. For LBW, both in all deliveries and in term deliveries only, there was no difference in the odds with increasing B-Hg tertile in the univariate or multivariate models (Table 3).

### 3.3 Regression models for B-Hg and birth outcomes stratified by fish-eating

When the regression models were repeated after stratification for fish-consumption or non-consumption, there were no significant associations of birth weight, head circumference or crown–heel length with B-Hg in fish-eaters or non-fish-eaters, with the exception of a negative association with birth weight in the adjusted model for non-fish-eaters (unstandardised regression coefficient B (g) = –58.39 (95% CI −113.81, −2.97, p = 0.039) (Table 2). There was a protective effect of B-Hg in fish-eaters on the odds of preterm delivery in univariate analysis that was attenuated on adjustment. There were no associations of B-Hg on the odds of LBW (in all deliveries and in term deliveries only) in fish-eaters and in non-fish-eaters in univariate and multivariate models (Table 3). Repetition of the models with further stratification into oily-fish-eaters and non-oily-fish-eaters did not show any associations in either group with birth weight, head circumference or crown–heel length and maternal B-Hg (Supplementary Table A4). In logistic analyses with tertiles of B-Hg, the odds of LBW for oily-fish-eaters was increased in tertiles 2 and 3 (reference tertile 1), but the confidence intervals were wide (Supplementary Table A5). This increase in odds was not apparent when those with preterm births were excluded from the LBW group (model failed to converge) (Supplementary Table A5).

### 3.4 Regression models with additional adjustment for B-Se

Repetition of the model 1 with additional adjustment for B-Se (model 2) did not alter any of the findings from model 1 (Table 2, Table 3; Supplementary Tables A4 and A5), with the exception of slightly weakening the association between B-Hg and birth weight in non-fish-eaters (unstandardised regression coefficient B (g) = –57.00 (95% CI − 112.51, −1.49), p = 0.044 (Table 3)).

## 4 Discussion

We found evidence for associations of B-Hg with increases in birth weight, head circumference and crown–heel length in univariate analyses, but these were attenuated in adjusted models. Similarly there was evidence for a weak protective effect on the odds of having a preterm birth with tertiles of B-Hg in univariate analysis, but no significant association after adjustment. There was no evidence for a change in the odds of having a LBW baby with increasing tertiles of B-Hg in univariate or multivariate analyses. Despite the fish-eaters having a B-Hg 32% higher than non-fish-eaters, there was no evidence that they had worse birth outcomes than non-fish-eaters. Finally, there was no evidence that B-Se had any effect on the outcomes of the models of B-Hg and birth outcomes.

Our study adds weight to the small but growing number of studies with a moderate level of maternal or cord B-Hg that have generally shown no associations of B-Hg with a range of birth outcomes (Supplementary Table A1). This is particularly the case in those studies in which maternal B-Hg is below the US level of concern of 5.8 μg/l for pregnant women (Committee on the Toxological Effects of Methylmercury, Board on Environmental Studies and Toxicology, National Research Council, 2000) (there is no published UK level of concern), as in our study. Lederman et al. (2008) and Gundacker et al. (2010), for example, found no associations with maternal B-Hg for these three outcomes with B-Hg of 1.6 (geometric mean, 95% CI 1.4, 1.8) μg/l and 0.7 (median) μg/l, respectively. Although it might be expected that associations would be more apparent for maternal blood or cord blood Hg above 5.8 μg/l this has not been consistently shown. For example, Lucas et al. (2004) found no association of birth weight or gestation age with geometric cord blood Hg of 14.1 (95% CI 13.1, 15.2) μg/l. At a particularly high mean cord blood Hg of 53.3 μg/l, however, Ramirez et al. (2000) found negative associations with head circumference and gestational age. Studies using other tissues to assess fetal Hg exposure make interpretation rather more difficult, but in general there has been little evidence to support any associations with birth outcomes (Supplementary Table A1). Taken together, the evidence is not greatly suggestive of a dose–response relationship of blood or cord Hg on birth outcomes except possibly at very high levels, but requires further studies across a range of blood or cord Hg to investigate fully.

These associations, however, may be confounded by fish consumption, in which nutrients provided by fish, such as polyunsaturated fatty acids, iodine, selenium and vitamin D, have a beneficial effect on birth outcomes. In the present study, after stratification for overall fish consumption we did not find any evidence that outcomes were different depending on whether the mother was a fish-eater or not. However, there was some evidence that B-Hg was negatively associated with birth weight in non-fish-eaters, suggesting that fish-eating might be protective against this association. Several studies have investigated the association of birth outcomes directly with fish consumption rather than with maternal Hg levels: Rogers et al. (2004) found a deceased risk of intrauterine growth retardation (birthweight <10th percentile) with increasing maternal fish (lean plus oily) intake in the UK ALSPAC cohort; in Denmark, however, increasing consumption of fatty fish was associated with a higher risk of the baby having a birth weight <10th percentile (Halldorsson et al., 2007). This difference may reflect the detrimental presence of pollutants such as organochlorine compounds that accumulate in the fat tissues of large oily fish accounting for differences in the effects of oily and lean fish, and a combination thereof. Many studies on maternal Hg exposure and birth outcomes do not report fish intake at all (Al-Saleh et al., 2014b; Hu et al., 2015, Lucas et al., 2004, Ramirez et al., 2000, Wells et al., 2016), while others have reported fish intake but have not used the data for adjustment in the models or stratification (Ding et al., 2013; Gundacker et al., 2010, Lee et al., 2010). Lack of adjustment for fish intake is likely to result in an underestimation of the toxic effects of Hg as well as the nutritional benefits of eating fish. Three studies that have adjusted for fish consumption in pregnancy have provided conflicting results (Al-Saleh et al., 2014a, Lederman et al., 2008, Ramon et al., 2009) (Supplementary Table A1). These studies, however, lacked detail in the method of collection of dietary data or in the types of fish consumed, specifically oily fish, which may have a relatively higher mercury content than white fish (US Food and Drug Administration, 2010). After stratification into oily-fish-eaters and non-oily-fish-eaters in the present study, we did not find any evidence for adverse effects of oily fish, despite maternal B-Hg being higher in oily-fish-eaters than non-fish-eaters. In the most comprehensive study to date to our knowledge on maternal Hg exposure, fish consumption and birth outcomes, Ramon et al. (2009) analysed cord blood from 554 infants born in Valencia, Spain, and obtained detailed data on consumption of different categories of fish: canned tuna/lean fish/large oily fish/small oily fish. The geometric mean cord blood Hg was relatively high (9.4 (95% CI 8.8, 10.2) μg/l; about 70% > 5.8 μg/l), reflecting high levels of maternal fish consumption. Both cord Hg and consumption of large oily fish were associated with some adverse birth outcomes, whereas canned tuna and lean fish were associated positively. Higher cord Hg was associated with reduced birth weight and more weakly with reduced birth length, and with an increased risk of SGA for length but not SGA for weight. The difference in findings in this study and in our study may reflect differences in the total amount of fish consumed as well as country-specific differences in the pollutant levels in fish. This requires further detailed investigation in other cohorts.

As recognised by the European Food Safety Authority(2015) and the Food and Agriculture Organization of the United Nations and World Health Organization (2011), guidelines on fish consumption inevitably differ by country depending on local conditions and differences in the species of fish consumed. The UK Food Standards Agency through NHS Choices issues advice to pregnant and breast-feeding women, and women planning to become pregnant, on fish consumption related to mercury, and to polychlorinated biphenyls (PCBs) and dioxin exposure (NHS Choices, 2015). With regard to mercury, the guidelines for pregnant women and women planning to become pregnant include three fish to avoid completely (predatory species) as well as information on fish for which intake should be limited, such as fresh and canned tuna. There are additional limitations advised with regard to PCBs and dioxins, adding to a rather complicated set of advice. The US guidelines are broadly similar (US Food and Drug Administration, 2004, US Food and Drug Administration, 2014), and it has been suggested that many pregnant women limit or avoid fish altogether in the face of the US guidelines (Oken et al., 2003, Shimshack and Ward, 2010). It is possible that the UK public health message to eat at least two portions of fish per week, at least one of which should be oily, during pregnancy is being lost, and this is likely to result in avoidance of fish and a concomitant loss of the beneficial effects of fish-eating on offspring.

There are several strengths of this study. (1) The study involved large numbers of pregnant women with both prenatal B-Hg and birth outcomes. (2) The study included a large number of participants who did not eat fish during pregnancy, in contrast to other studies on the health outcomes associated with Hg which are often conducted in high-fish-eating populations (e.g. Seychelles Study, Project Viva). (3) The study included data on the type of fish consumed enabling stratification into oily-fish-eaters and non-oily-fish-eaters. (4) The prenatal exposure was B-Hg measured in the first half of pregnancy, in contrast to studies which have relied on cord blood Hg or other Hg in other matrices such as hair, urine or diet. There are also a number of limitations. (1) Although we were able to account for many possible confounders in our analyses, there are likely to be others that were unable to adjust for. These include, for example, pollutant levels in fish such as organochlorine compounds. (2) The FFQ was completed in the third trimester of pregnancy, sometime after collection of the blood sample for Hg and Se analysis. However, it has been shown that the dietary patterns of these women remain fairly constant (Northstone and Emmett, 2008), so it is likely that patterns of fish consumption were maintained.

## 5 Conclusion

Moderate mercury levels in pregnancy were not associated with effects on anthropometric variables, or on the risk of preterm birth or LBW, in this study. There was some evidence to suggest a detrimental effect of maternal B-Hg on birthweight in non-fish-eaters that was not apparent in fish-eaters. This suggests that fish consumption may have a protective effect on birthweight. Consumption of fish other than high level predatory feeders in line with government guidelines during pregnancy (at least two portions of fish per week, one of which should be oily) should be encouraged.

## Competing interests

The authors declare they have no conflicts of interest.

## Figures and Tables

**Table 1:** Maternal blood Hg and Se levels by fish consumption from a food frequency questionnaire during pregnancy in women in the ALSPAC study.

	Never or rarely	Once in 2 weeks	1–3 times per week	4–7 times per week/More than once per day	P value
B-Hg					
White fish	1.63 ± 1.02 (n = 643)	2.09 ± 0.99 (n = 1392)	2.35 ± 1.14 (n = 1372)	2.34 ± 1.15 (n = 49)	<0.001
Oily fish	1.75 ± 0.94 (n = 1474)	2.28 ± 1.07 (n = 1138)	2.52 (n = 802)	2.38 ± 1.02 (n = 42)	<0.001

B-Se					
White fish	108.98 ± 24.45 (n = 643)	112.45 ± 24.02 (n = 1392)	114.64 ± 24.14 (n = 1372)	114.60 ± 25.53 (n = 49)	<0.001
Oily fish	106.91 ± 23.20 (n = 1474)	114.70 ± 22.89 (n = 1138)	119.30 ± 25.60 (n = 802)	119.89 ± 25.34 (n = 42)	<0.001

Values are mean ± SD (ANOVA).

**Table 2:** Linear regression analysis: maternal B-Hg (μg/l) as a predictor of birth outcomes in women in ALSPAC.

	n	R^2^	Unstandarised B regression coefficient	95% CI	p value
All					
Birth weight (g)					
Univariate	3853	0.001	17.86	0.80, 33.9	**0.029**
Multivariate model 1	2692	0.378	−3.05	−18.87, 12.77	0.705
Multivariate model 2	2693	0.378	−4.15	−20.49, 12.20	0.619
Head circumference (cm)					
Univariate	3342	0.001	0.05	0.00, 0.10	**0.033**
Multivariate model 1	2376	0.321	0.01	−0.04, 0.06	0.628
Multivariate model 2	2376	0.324	0.01	−0.04, 0.05	0.853
Crown–heel (cm)					
Univariate	3297	0.002	0.10	0.03, 0.17	**0.005**
Multivariate model 1	2345	0.315	0.02	−0.06, 0.09	0.637
Multivariate model 2	2345	0.315	0.01	−0.07, 0.09	0.743
**–**					
**Fish eaters**					
Birth weight (g)					
Univariate	2923	0.000	4.25	−13.46, 21.97	0.638
Multivariate model 1	2324	0.354	−1.46	−18.58, 15.65	0.867
Multivariate model 2	2324	0.354	−3.28	−21.04, 14.48	0.717
Head circumference (cm)					
Univariate	2585	0.001	0.03	−0.02, 0.08	0.206
Multivariate model 1	2055	0.320	0.01	−0.04, 0.06	0.628
Multivariate model 2	2055	0.321	0.00	−0.05, 0.05	0.956
Crown–heel (cm)					
Univariate	2548	0.001	0.06	−0.03, 0.14	0.179
Multivariate model 1	2026	0.305	0.02	−0.07, 0.10	0.709
Multivariate model 2	2026	0.305	0.01	−0.08, 0.09	0.893
**Non-fish-eaters**					
Birth weight (g)					
Univariate	500	0.000	−4.16	−54.43, 46.12	0.871
Multivariate model 1	354	0.408	−58.39	−113.81, −2.97	**0.039**
Multivariate model 2	354	0.409	−57.00	−112.51, −1.49	**0.044**
Head circumference (cm)					
Univariate	439	0.000	0.00	−0.16, 0.16	0.998
Multivariate model 1	311	0.311	−0.05	−0.24, 0.15	0.630
Multivariate model 2	311	0.311	−0.05	−0.24, 0.15	0.634
Crown–heel (cm)					
Univariate	437	0.001	0.08	−0.16, 0.31	0.523
Multivariate model 1	310	0.397	−0.08	−0.33, 0.17	0.540
Multivariate model 2	310	0.399	−0.08	−0.33, 0.17	0.551

Model 1: Adjusted for maternal educational attainment, age, parity, height, BMI, sex of baby, gestational age at delivery, smoking, alcohol consumption.

Model 2: Model 1 + maternal selenium.

**Table 3:** Logistic regression analysis: tertiles of maternal B-Hg as a predictor of preterm delivery and of LBW in women in ALSPAC.

	All			Stratified by fish-eating					
				Fish-eaters			Non-fish-eaters		
	OR (95% CI)	P value	p trend	OR (95% CI)	P value	p trend	OR (95% CI)	P value	p trend
Preterm (reference Not preterm)									
Univariate	N = 3895			N = 2951			N = 505		
Tertile 1	Ref	–	0.006	Ref	–	0.014	Ref	–	0.38
Tertile 2	0.67 (0.48, 0.94)	0.021		0.58 (0.38, 0.89)	0.012		0.93 (0.30, 3.00)	0.907	
Tertile 3	0.62 (0.44, 0.88)	0.007		0.59 (0.39, 0.89)	0.012		0.37 (0.05, 2.89)	0.339	
Multivariate model 1^a^	N = 2720			N = 2349			N = 357		
Tertile 1	Ref	–	0.061	Ref	–	0.071	Ref	–	0.661
Tertile 2	0.69 (0.44, 1.08)	0.105		0.66 (0.40, 1.07)	0.09		1.18 (0.29, 4.82)	0.818	
Tertile 3	0.64 (0.40, 1.03)	0.064		0.62 (0.37, 1.03)	0.065		0.51 (0.06, 4.42)	0.538	
Multivariate model 2^a^	N = 2720			N = 2720			N = 357		
Tertile 1	Ref	–	0.067	Ref	–	0.096	Ref	–	0.668
Tertile 2	0.69 (0.44, 1.08)	0.107		0.66 (0.41, 1.08)	0.101		1.21 (0.29, 4.99)	0.792	
Tertile 3	0.64 (0.40, 1.04)	0.071		0.64 (0.40, 1.04)	0.089		0.51 (0.06, 4.41)	0.538	
LBW (reference Not LBW)									
Univariate	N = 3853			N = 2923			N = 500		
Tertile 1	Ref	–	0.117	Ref	–	0.518	Ref	–	0.784
Tertile 2	0.80 (0.55, 1.16)	0.246		0.78 (0.48, 1.27)	0.32		1.23 (0.48, 3.50)	0.605	
Tertile 3	0.74 (0.51, 1.08)	0.12		0.84 (0.52, 1.35)	0.47		1.06 (0.30, 3.84)	0.926	
Multivariate model 1^b^	N = 2692			N = 2324			N = 354		
Tertile 1	Ref	–	0.789	Ref	–	0.521	Ref	–	0.38
Tertile 2	1.14 (0.69, 1.90)	0.605		1.73 (0.85, 3.53)	0.134		1.16 (0.23, 5.85)	0.86	
Tertile 3	1.08 (0.63, 1.84)	0.783		1.35 (0.64, 2.86)	0.437		2.83 (0.37, 21.53)	0.313	
Multivariate model 2^b^	N = 2692			N = 2324			N = 354		
Tertile 1	Ref	–	0.275	Ref	–	0.371	Ref	–	0.391
Tertile 2	1.73 (0.93, 3.24)	0.086		1.81 (0.88, 3.74)	0.106		1.08 (0.20, 5.74)	0.924	
Tertile 3	1.46 (0.74, 2.89)	0.275		1.50 (0.68, 3.27)	0.314		2.87 (0.38, 21.80)	0.309	
LBW in term deliveries (reference Not LBW in term deliveries)									
Univariate	N = 3656			N = 2787			N = 283		
Tertile 1	Ref	–	0.709	Ref	–	0.718	Ref	–	0.322
Tertile 2	1.30 (0.72, 2.33)	0.382		1.47 (0.71, 3.04)	0.294		0.92 (0.18, 4.64)	0.922	
Tertile 3	0.89 (0.47, 1.67)	0.712		0.93 (0.43, 2.02)	0.86		2.30 (0.56, 9.44)	0.247	
Multivariate model 1^b^	N = 2569			N = 2218			N = 341		
Tertile 1	Ref	–	0.666	Ref	–	0.926	Ref	–	0.437
Tertile 2	2.12 (0.96, 4.70)	0.065		2.46 (0.96, 6.35)	0.062		0.39 (0.03, 5.34)	0.483	
Tertile 3	1.23 (0.54, 3.13)	0.564		1.27 (0.45, 3.60)	0.654		3.19 (0.42, 24.55)	0.265	
Multivariate model 2^b^	N = 2569			N = 2218			N = 341		
Tertile 1	Ref	–	0.678	Ref	–	0.776	Ref	–	0.376
Tertile 2	2.11 (0.95, 4.70)	0.067		2.59 (1.00, 6.76)	0.051		0.51 (0.04, 6.91)	0.616	
Tertile 3	1.29 (0.52, 3.16)	0.582		1.40 (0.48, 4.09)	0.544		3.32 (0.42, 26.05)	0.254	

Preterm, <37 weeks; LBW, <2500 g.

a Model 1: Adjusted for maternal educational attainment, age, parity, height, BMI, sex of baby, smoking, alcohol consumption; Model 2: Model 1 + maternal selenium.

b Model 1: Adjusted for maternal educational attainment, age, parity, height, BMI, sex of baby, gestational age at delivery, smoking, alcohol consumption; Model 2: Model 1 + maternal selenium.
