# Supplementary material for: Blood mercury levels and fish consumption in pregnancy: Risks and benefits for birth outcomes in a prospective observational birth cohort
Source: Int J Hyg Environ Health. 2016 Aug;219(6):513–20. doi: 10.1016/j.ijheh.2016.05.004 (PMC4970655; doi:10.1016/j.ijheh.2016.05.004)

**Supplementary Table A1**Summary of studies modelling associations of mercury levels in pregnancy on birth outcomes

| **Exposure measure** | **Reference** | **Sample size** | **Location** | **Exposure level** | **Above or below US level of concern** | **Seafood intake** | **Se levels** | **Associations** | | | |
| --- | --- | --- | --- | --- | --- | --- | --- | --- | --- | --- | --- |
|  |  |  |  |  |  |  |  | **Birth weight** | **Birth length/CHL** | **Head circumference** | **Gestational age/preterm** |
| Maternal blood | Taylor et al. (present study) | 4044 | UK | Mean 2.07 (SD 1.10), range 0.17–12.76 µg/l | Below | Stratified by fish intake | Adjusted for Se level | No association | No association | No association | No association |
|  |  |  |  |  |  |  |  |  |  |  |  |
|  | Hu et al. (2015) ^a^ | 81 | China | NR | Below | - | - | No association | - | - | - |
|  | Ding et al. (2013) | 258 | China | GM 0.83 µg/l, mean 0.91 (SD 0.37) | Below | Reported fish intake | - | No association | No association | No association | - |
|  | Al-Saleh et al. (2014b) | 1578 | Saudi Arabia | Mean 3.01 (SD 6.32), median 1.95, range 0.00  –206.41 µg/l | Below | - | - | No association | No association | No association | - |
|  | Lee et al. (2010) | 417 | South Korea | GM 5.53, range 0.23  –24.1 µg/l | Below | Reported fish intake | - | -ve association | - | - | - |
|  | Lederman et al. (2008) | 329 | USA | GM 1.6 (95% CI 1.4, 1.81) µg/l | Below | Adjusted for seafood intake | - | No association | No association | No association | No association |
|  | Gundacker et al. (2010) | 53 | Austria | Range 0.1  –5.2, median 0.7 µg/l | Below | Reported fish intake | - | No association | No association | No association | - |
|  | Foldspang and Hansen (1990)^c^ | 376 | Greenland | Mean 14.9 (range 2  –128) µg/l | Above | Adjusted for marine food intake^b^ | - | -ve association | - | - | No association |
|  | Ramirez et al. (2000) ^a^ | 78 | The Philippines | Mean 24 (SD 5.5) µg/l | Above | - | - | - | - | No association | No association |
| Cord blood | Wells et al. (2016) | 271 | USA | GM MeHg 0.13 (95% CI 0.10–0.17) µg/l | [Below] | - | - | No association | No association | No association | No association |
|  | Hu et al. (2015)^a^ | 81 | China | NR | - | - | - | No association | - | - | - |
|  | Al-Saleh et al. (2014b) | 1578 | Saudi Arabia | Mean 3.35 (SD 2.67), median 2.88, range 0.00–26.53 µg/l | [Below] | - | - | No association | No association | No association | - |
|  | Al-Saleh et al. (2014a) | 250 | Saudi Arabia | Mean 3.46 (SD 2.51), median 3.15, range 0.00  –15.52 µg/l | [Below] | Adjusted for fish intake | Adjusted for Se levels | -ve association Increased risk of SGA | +ve association | -ve association | - |
|  | Ding et al. (2013) | 258 | China | GM 1.46 µg/l, mean 1.60 (SD 0.72) | [Below] | Reported fish intake | - | No association | No association | No association |  |
|  | Lee et al. (2010) | 417 | South Korea | GM 5.53, range 0.23–24.1 µg/l | [Below] | Reported fish intake | - | -ve association | - | - | - |
|  | Ramon et al. (2009) | 554 | Spain | GM 9.4 (95% CI 8.8, 10.2) µg/l | [Above] | Adjusted for type of fish consumed | - | -ve association  (canned tuna +ve association) Increased risk of SGA for length  Oily fish associated with increased risk SGA for weight  Lean fish associated with lower risk SGA for length | No association | - | - |
|  | Gundacker et al. (2010) | 53 | Austria | Range 0.2  –6.8 µg/l | [Below] | Reported fish intake | - | No association | No association | No association | - |
|  | Lucas et al. (2004) | 439 | Canada | GM 14.1 µg/l (95% CI 13.1, 15.2) µg/l | [Above] | - | - | No association | - | - | No association |
|  | Ramirez et al. (2000) | 78 | The Philippines | Mean 53.3 (SD 37.49) µg/l | [Above] | - | - | - | - | -ve association | Adverse association with gestational age |
| Cord tissue | Daniels et al. (2007) | 1040 | UK | Median 0.01 µg/g wet weight | - | Adjusted for fish intake | - | No association | - | - | No association |
| Hair | Marques et al. (2013) | 1433 | Brazil | Riverine: median 12.12 µg/g  Urban: median 5.36 µg/g  Rural: median 7.82 µg/g  Tin mining: median 4.45 µg/g | - | Reported fish intake | - | No association | - | - |  |
|  | Drouillet-Pinard et al. (2010) | 645 | France | Median 0.52 (IQR 0.30  –0.82) µg/g | - | Adjusted for seafood intake | - | No association | No association | No association | No association |
|  | Gundacker et al. (2010) | 53 | Austria | Range 0.05-0.77 µg/l | - | Reported fish intake | - | No association | No association | No association | - |
|  | Xue et al. (2007) | 1024 | USA | Mean 0.29, median 0.23, range 0.01  –2.50  ≥90th percentile 0.55-2.50 µg/g | - | Adjusted for fish intake | - | - | - | - | Adverse association with preterm |
|  | Sikorski et al. (1986) | 329 | Poland | Maternal scalp hair: mean 0.11, range nd-0.62 µg/g | - | - | - | No association | No association | No association | - |
|  |  |  |  | Maternal pubic hair: mean 1.01, range nd-31.86 µg/g | - | - | - | No association | No association | No association | - |
|  |  |  |  | Infant hair: mean 1.88, range 0.02  –40.6 µg/g | - | - | - | -ve association | No association | No association | - |
| Diet Hg in first 5 months of pregnancy | Vejrup et al. (2014) | 62,941 | Norway | Mean 0.14 mg/kg body weight | - | Stratified by fish intake | - | -ve association | - | - | Adverse association with gestational age |
| Creatinine-corrected maternal urine | Bashore et al. (2014) | 191 | USA | - | - | Reported seafood intake | - | No association | No association | No association | No association |
| Meconium | Ramirez et al. (2000) | 78 | The Philippines | Mean 48.6±43.48 µg/g | - | - |  | -ve association | - | -ve association | - |
|  | Gundacker et al. (2010) | 53 | Austria | Range 0.4-128 µg/g | - | Reported fish intake | - | No association | No association | No association | - |
| Breast milk | Gundacker et al. (2010) | 53 | Austria | Range 0.1-2.0 [unit?] | - | Reported fish intake | - | No association | No association | No association | - |
| Placenta | Al-Saleh et al. (2014b) | 1578 | Saudi Arabia | Mean 0.06 (SD 0.40), median 0.03, range 0.000-13.00 µg/g dry wt | - | - | - | No association | -ve association | No association | - |
|  | Al-Saleh et al. (2014a) | 250 | Saudi Arabia | Mean 0.06 (SD 0.08), median 0.03, range 0-0.78 µg/g dry wt | - | Adjusted for fish intake | Adjusted for Se levels | -ve association | -ve association | No association | No association |
|  | Gundacker et al. (2010) | 53 | Austria | Range 0.1–11.7 µg/kg | - | Reported fish intake | - | No association | No association | No association | - |

Adapted and extended from Karagas et al. (2012).

^a^Abstract only.

^b^Primarily whale and seal meat.

^c^Results refuted on reanalysis (Bjerregaard and Hansen 2000).

CHL, crown–heel length; GM, geometric mean; IQR, interquartile range; nd, not detected; NR, not reported; SGA, small for gestational age.

**References**

Al-Saleh I, Al-Rouqi R, Obsum CA, Shinwari N, Mashhour A, Billedo G, et al. 2014a. Mercury (Hg) and oxidative stress status in healthy mothers and its effect on birth anthropometric measures. Int J Hyg Environ Health 217:567-585.

Al-Saleh I, Shinwari N, Mashhour A, Rabah A. 2014b. Birth outcome measures and maternal exposure to heavy metals (lead, cadmium and mercury) in Saudi Arabian population. Int J Hyg Environ Health 217:205-218.

Bashore CJ, Geer LA, He X, Puett R, Parsons PJ, Palmer CD, et al. 2014. Maternal mercury exposure, season of conception and adverse birth outcomes in an urban immigrant community in Brooklyn, New York, U.S.A. Int J Environ Res Public Health 11:8414-8442.

Bjerregaard P, Hansen JC. 2000. Organochlorines and heavy metals in pregnant women from the Disko Bay area in Greenland. Sci Total Environ 245:195-202.

Daniels JL, Rowland AS, Longnecker MP, Crawford P, Golding J, Team AS. 2007. Maternal dental history, child's birth outcome and early cognitive development. Paediatr Perinatal Epidemiol 21:448-457.

Ding G, Cui C, Chen L, Gao Y, Zhou Y, Shi R, et al. 2013. Prenatal low-level mercury exposure and neonatal anthropometry in rural Northern China. Chemosphere 92:1085-1089.

Drouillet-Pinard P, Huel G, Slama R, Forhan A, Sahuquillo J, Goua V, et al. 2010. Prenatal mercury contamination: Relationship with maternal seafood consumption during pregnancy and fetal growth in the 'Eden mother-child' cohort. Br J Nutr 104:1096-1100.

Foldspang A, Hansen JC. 1990. Dietary intake of methylmercury as a correlate of gestational length and birth weight among newborns in Greenland. Am J Epidemiol 132:310-317.

Gundacker C, Frohlich S, Graf-Rohrmeister K, Eibenberger B, Jessenig V, Gicic D, et al. 2010. Perinatal lead and mercury exposure in austria. Sci Total Environ 408:5744-5749.

Hu X, Zheng T, Cheng Y, Holford T, Lin S, Leaderer B, et al. 2015. Distributions of heavy metals in maternal and cord blood and the association with infant birth weight in china. J Reprod Med 60:21-29.

Karagas MR, Choi AL, Oken E, Horvat M, Schoeny R, Kamai E, et al. 2012. Evidence on the human health effects of low-level methylmercury exposure. Environ Health Perspect 120:799-806.

Lederman SA, Jones RL, Caldwell KL, Rauh V, Sheets SE, Tang D, et al. 2008. Relation between cord blood mercury levels and early child development in a world trade center cohort. Environ Health Perspect 116:1085-1091.

Lee BE, Hong YC, Park H, Ha M, Koo BS, Chang N, et al. 2010. Interaction between GSTM1/GSTT1 polymorphism and blood mercury on birth weight. Environ Health Perspect 118:437-443.

Lucas M, Dewailly E, Muckle G, Ayotte P, Bruneau S, Gingras S, et al. 2004. Gestational age and birth weight in relation to n-3 fatty acids among Inuit (Canada). Lipids 39:617-626.

Marques RC, Bernardi JV, Dorea JG, Brandao KG, Bueno L, Leao RS, et al. 2013. Fish consumption during pregnancy, mercury transfer, and birth weight along the madeira river basin in Amazonia. Int J Environ Res Public Health 10:2150-2163.

Ramirez GB, Cruz MC, Pagulayan O, Ostrea E, Dalisay C. 2000. The Tagum study i: Analysis and clinical correlates of mercury in maternal and cord blood, breast milk, meconium, and infants' hair. Pediatrics 106:774-781.

Ramon R, Ballester F, Aguinagalde X, Amurrio A, Vioque J, Lacasana M, et al. 2009. Fish consumption during pregnancy, prenatal mercury exposure, and anthropometric measures at birth in a prospective mother-infant cohort study in Spain. Am J Clin Nutr 90:1047-1055.

Sikorski R, Paszkowski T, Szprengier-Juszkiewicz T. 1986. Mercury in neonatal scalp hair. Sci Total Environ 57:105-110.

Vejrup K, Brantsaeter AL, Knutse HK, Magnus P, Alexander J, Kvalem HE, et al. 2014. Prenatal mercury exposure and infant birth weight in the Norweigan mother and child cohort study. Public Health Nutr 17:2071-2080.

Wells EM, Herbstman JB, Lin YH, Jarrett J, Verdon CP, Ward C, et al. 2016. Cord blood methylmercury and fetal growth outcomes in Baltimore newborns: Potential confounding and effect modification by omega-3 fatty acids, selenium, and sex. Environ Health Perspect 124:373-9.

Xue F, Holzman C, Rahbar MH, Trosko K, Fischer L. 2007. Maternal fish consumption, mercury levels, and risk of preterm delivery. Environ Health Perspect. 115:42-47.

**Supplementary Table A2**  Characteristics of participants in ALSPAC included and excluded

|  | **Included** | | **Excluded** | | **P value** |
| --- | --- | --- | --- | --- | --- |
| Maternal characteristic | **n** |  | n |  |  |
| Blood Hg (µg/l) | 4044 | 2.07±1.10 (0.17–12.76) | 90 | 2.08±1.27 (0.52-6.73) | 0.944 |
| Parity (n) |  |  |  |  |  |
| 0 | 3595 | 1601 (14.6%) | 60 | 23 (38.3%) | 0.362 |
| ≥1 |  | 1994 (85.4%) |  | 37 (61.7%) |  |
| BMI (kg/m^2^) | 3301 | 23.0±3.8 (15.4–51.2) | 56 | 23.2 4.4 (28.7-39.7) |  |
| Age (years) | 3696 | 28.0±4.98 (15–45) | 69 | 28.8±4.16 (21-40) | 0.138 |
| Education |  |  |  |  |  |
| None/CSE/Vocational/O Level | 3505 | 2159 (61.5%) | 52 | 32 (61.5%) | 0.914 |
| A level |  | 800 (22.8%) |  | 12 (23.1%) |  |
| Degree |  | 546 (15.6%) |  | 8 (15.4%) |  |
| Smoker |  |  |  |  |  |
| Yes | 3572 | 742 (20.8%) | 61 | 14 (23.0%) | 0.556 |
| No |  | 2830 (79.2%) |  | 47 (77.0%) |  |
| Ate fish |  |  |  |  |  |
| Yes | 3556 | 2951 (85.4%) | 52 | 48 (92.3%) | 0.231 |
| No |  | 505 (14.6%) |  | 4 (7.7%) |  |

Values are mean±SD or n (%).

**Supplementary Table A3** Characteristics of all pregnant women with a blood Hg measurement in ALSPAC and stratified by fish-eating in pregnancy

|  | **All** | | **Stratified by fish-eating** | | | | |
| --- | --- | --- | --- | --- | --- | --- | --- |
|  |  |  | **Fish eaters** | | **Non-fish eaters** | | **P value for fish eater vs non-fish eater** |
| Mother |  | n |  | n |  | n |  |
| Blood Hg (µg/l) | 2.07±1.10 (0.17–12.76) | 4044 | 2.21±1.08 (0.17–11.54) | 2951 | 1.52±0.96 (0.17–12.10) | 505 | <0.001 |
| Parity (n) |  |  |  |  |  |  |  |
| 0 | 1601 (14.6%) | 3595 | 1250 (44.4) | 2951 | 224 (46.9%) | 505 | 0.318 |
| ≥1 | 1994 (85.4%) |  | 1565 (55.6) |  | 254 (53.1%) |  |  |
| BMI (kg/m^2^) | 23.0±3.8 (15.4–51.2) | 3301 | 23.0±3.8 (15.4–51.2) | 2815 | 23.1±4.0 (15.6–41.1) | 478 | 0.643 |
| Age (years) | 28.0±4.98 (15–45) | 3696 | 28.4±4.7 (15–44) | 2880 | 26.7 (15–42) | 484 | <0.001 |
| Education |  |  |  |  |  |  |  |
| None/CSE/Vocational/O Level | 2159 (61.5%) | 3505 | 1751 (59.7%) | 2880 | 352 (71.0%) | 484 | <0.001 |
| A level | 800 (22.8%) |  | 694 (23.7%) |  | 93 (18.8%) |  |  |
| Degree | 546 (15.6%) |  | 486 (16.6%) |  | 51 (10.3%) |  |  |
| Smoker |  |  |  |  |  |  |  |
| Yes | 742 (20.8%) | 3572 | 505 (17.1%) | 2931 | 143 (30.0%) | 496 | <0.001 |
| No | 2830 (79.2%) |  | 2328 (82.2%) |  | 333 (70.0%) |  |  |
| Ate fish |  |  |  |  |  |  |  |
| Yes | 2951 (85.4%) | 3556 | 2951 (100%) | 2833 | 0 | 476 | - |
| No | 505 (14.6%) |  | 0 |  | 505 (100%) |  |  |
|  |  |  |  |  |  |  |  |
| Offspring |  |  |  |  |  |  |  |
| Gestational age (weeks) | 39.5±1.9 (25.0–46.0) | 3895 | 39.6±1.71 (26.0–45.0) | 2951 | 39.5±1.6 (29.0–44.0) | 505 | 0.597 |
| Birthweight (g) | 3426.4±555.2 (550–5640) | 3853 | 3456.8±527.6 (815–5640) | 2923 | 3369.2±546.1 (910–5080) | 500 | 0.001 |
| Head circumference (cm) | 34.8±1.5 (20.9–40.0) | 3342 | 34.9±1.4 (20.9–39.8) | 2585 | 34.7±1.6 (20.9–40.0) | 439 | 0.020 |
| Crown–heel length (cm) | 50.7±2.3 (36.0–61.0) | 3297 | 50.8±2.3 (40.1–61.0) | 2548 | 50.4±2.4 (37.1–57.0) | 437 | 0.001 |
| Preterm |  |  |  |  |  |  |  |
| Yes | 199 (4.9%) | 3895 | 138 (4.7%) | 2951 | 17 (3.4%) | 505 | 0.189 |
| No | 3696 (91.4%) |  | 2813 (95.3%) |  | 488 (96.6%) |  |  |
| LBW |  |  |  |  |  |  |  |
| Yes | 167 (4.3%) | 3853 | 108 (3.7%) | 2923 | 22 (4.4%) | 500 | 0.446 |
| No | 3686 (95.7%) |  | 2815 (95.4%) |  | 478 (95.6%) |  |  |
| LBW in term deliveries only |  |  |  |  |  |  |  |
| Yes | 66 (1.8%) | 3656 | 50 (1.7%) | 2787 | 11 (2.2%) | 483 | 0.265 |
| No | 3590 (98.2%) |  | 2737 (92.7%) |  | 472 (93.5%) |  |  |
|  |  |  |  |  |  |  |  |

Values are mean±SD (range in parentheses) or n (%).

Fish consumption ascertained by a food frequency questionnaire at 32 weeks’ gestation

**Supplementary Table A4**  Linear regression analysis: maternal B-Hg (µg/l) as a predictor of birth outcomes for oily-fish-eaters and non-oily-fish-eaters in ALSPAC

|  | **n** | **R^2^** | **Unstandarised B regression coefficient** | **95% CI** | **p value** |
| --- | --- | --- | --- | --- | --- |
| **Oily-fish-eaters** |  |  |  |  |  |
| Birth weight (g) |  |  |  |  |  |
| Univariate | 1963 | 0.001 | -2.10 | -23.05, 18.84 | 0.844 |
| Multivariate model 1 | 1569 | 0.350 | 0.18 | -17.87, 22.11 | 0.835 |
| Multivariate model 2 | 1569 | 0.350 | -0.66 | -21.32, 20.00 | 0.950 |
| Head circumference (cm) |  |  |  |  |  |
| Univariate | 1732 | 0.001 | 0.01 | -0.04, 0.08 | 0.563 |
| Multivariate model 1 | 1384 | 0.332 | 0.02 | -0.04, 0.08 | 0.564 |
| Multivariate model 2 | 1384 | 0.332 | 0.01 | -0.05, 0.07 | 0.684 |
| Crown–heel (cm) |  |  |  |  |  |
| Univariate | 1710 | 0.001 | 0.05 | -0.04, 0.15 | 0.267 |
| Multivariate model 1 | 1365 | 0.299 | 0.04 | -0.06, 0.14 | 0.409 |
| Multivariate model 2 | 1365 | 0.299 | 0.03 | -0.07, 0.13 | 0.543 |
| **Non-oily-fish-eaters** |  |  |  |  |  |
| Birth weight (g) |  |  |  |  |  |
| Univariate | 1460 | 0.000 | 9.42 | -19.64, 38.48 | 0.525 |
| Multivariate model 1 | 1109 | 0.376 | -25.58 | -54.60, 3.45 | 0.084 |
| Multivariate model 2 | 1109 | 0.376 | -23.91 | -53.54, 5.71 | 0.114 |
| Head circumference (cm) |  |  |  |  |  |
| Univariate | 1292 | 0.001 | 0.05 | -0.04, 0.13 | 0.279 |
| Multivariate model 1 | 982 | 0.300 | -0.01 | -0.10, 0.08 | 0.775 |
| Multivariate model 2 | 982 | 0.301 | -0.03 | -0.12, 0.07 | 0.598 |
| Crown–heel (cm) |  |  |  |  |  |
| Univariate | 1275 | 0.001 | 0.07 | -0.07, 0.21 | 0.323 |
| Multivariate model 1 | 971 | 0.340 | -0.04 | -0.18, 0.10 | 0.549 |
| Multivariate model 2 | 971 | 0.340 | -0.04 | -0.18, 0.10 | 0.585 |

Model 1: Adjusted for maternal educational attainment, age, parity, height, BMI, sex of baby, gestational age at delivery, smoking, alcohol consumption.

Model 2: Model 1 + maternal selenium.

**Supplementary Table A5**  Logistic regression analysis: tertiles of maternal B-Hg as a predictor of preterm delivery and of LBW for oily-fish-eaters and non-oily-fish-eaters in ALSPAC

|  | **Oily-fish-eaters** | | | **Non-oily-fish-eaters** | | |
| --- | --- | --- | --- | --- | --- | --- |
|  | **OR (95% CI)** | **P value** | **p trend** | **OR (95% CI)** | **P value** | **p trend** |
| Preterm (reference Not Preterm) |  |  |  |  |  |  |
| Univariate | N=1982 |  |  | N=1474 |  |  |
| Tertile 1 | Ref | - | 0.189 | Ref | -- | 0.076 |
| Tertile 2 | 0.69 (0.39, 1.21) | 0.196 |  | 0.63 (0.35, 1.13) | 0.118 |  |
| Tertile 3 | 0.67 (0.39, 1.15) | 0.146 |  | 0.60 (0.30, 1.18) | 0.135 |  |
| Multivariate model 1^a^ | N=1585 |  |  | N=1121 |  |  |
| Tertile 1 | Ref | - | 0.253 | Ref | 0.634 | 0.424 |
| Tertile 2 | 0.65 (0.33, 1.28) | 0.212 |  | 0.86 (0.45, 1.62) | 0.448 |  |
| Tertile 3 | 0.65 (0.33, 1.26) | 0.199 |  | 0.74 (0.34, 1.62) |  |  |
| Multivariate model 2^a^ | N=1585 |  |  | N=1121 |  |  |
| Tertile 1 | Ref | - | 0.295 | Ref | - | 0.400 |
| Tertile 2 | 0.66 (0.34, 1.29) | 0.224 |  | 0.85 (0.45, 1.61) | 0.615 |  |
| Tertile 3 | 0.66 (0.33, 1.31) | 0.237 |  | 0.72 (0.33, 1.60) | 0.424 |  |
|  |  |  |  |  |  |  |
| LBW (reference Not LBW) |  |  |  |  |  |  |
| Univariate | N=1963 |  |  | N=1460 |  |  |
| Tertile 1 | Ref | - | 0.540 | Ref | - | 0.241 |
| Tertile 2 | 1.47 (0.70, 3.06) | 0.308 |  | 0.59 (0.32, 1.12) | 0.105 |  |
| Tertile 3 | 1.35 (0.66, 2.78) | 0.409 |  | 0.75 (0.40, 1.47) | 0.407 |  |
| Multivariate model 1^b^ | N=1569 |  |  | N=1109 |  |  |
| Tertile 1 | Ref | - | 0.140 | Ref | - | 0.734 |
| Tertile 2 | 3.98 (1.30, 12.17) | 0.016 |  | 0.80 (0.33, 1.90) | 0.606 |  |
| Tertile 3 | 3.23 (1.03, 10.07) | 0.044 |  | 0.89 (0.32, 2.45) | 0.820 |  |
| Multivariate model 2^b^ | N=1569 |  |  | N=1109 |  |  |
| Tertile 1 | Ref | - | 0.097 | Ref | - | 0.772 |
| Tertile 2 | 6.54 (1.72, 24.80) | 0.006 |  | 0.80 (0.34, 1.91) | 0.615 |  |
| Tertile 3 | 4.66 (1.18, 18.33) | 0.028 |  | 0.92 (0.33, 2.26) | 0.865 |  |
|  |  |  |  |  |  |  |
| LBW in not preterm (reference Not LBW in not preterm) |  |  |  |  |  |  |
| Univariate | N=1878 |  |  | N=1392 |  |  |
| Tertile 1 | Ref | - | 0.720 | Ref | - | 0.543 |
| Tertile 2 | 3.22 (0.94, 11.00) | 0.062 |  | 0.74 (0.30, 1.84) | 0.513 |  |
| Tertile 3 | 1.93 (0.55, 6.77) | 0.303 |  | 0.77 (0.28, 2.16) | 0.623 |  |
| Multivariate model 1^b^ | M=1504 |  |  | N=1055 |  |  |
| Tertile 1 | Ref | - | - | Ref | - | 0.630 |
| Tertile 2 | -^c^ |  |  | 0.75 (0.26, 2.23) | 0.608 |  |
| Tertile 3 | - |  |  | 0.76 (0.23, 2.66) | 0.685 |  |
| Multivariate model 2^b^ | N=1504 |  |  | N=1055 |  |  |
| Tertile 1 | Ref | - | - | Ref | - | 0.554 |
| Tertile 2 | -^c^ |  |  | 0.73 (0.25, 2.17) | 0.572 |  |
| Tertile 3 | - |  |  | 0.72 (0.21, 2.49) | 0.605 |  |

^a^Model 1: Adjusted for maternal educational attainment, age, parity, height, BMI, sex of baby, smoking, alcohol consumption; Model 2: Model 1 + maternal selenium.

^b^Model 1: Adjusted for maternal educational attainment, age, parity, height, BMI, sex of baby, gestational age at delivery, smoking, alcohol consumption; Model 2: Model 1 + maternal selenium.

^c^Models failed to converge.

Preterm, <37 weeks; LBW, <2500 g.


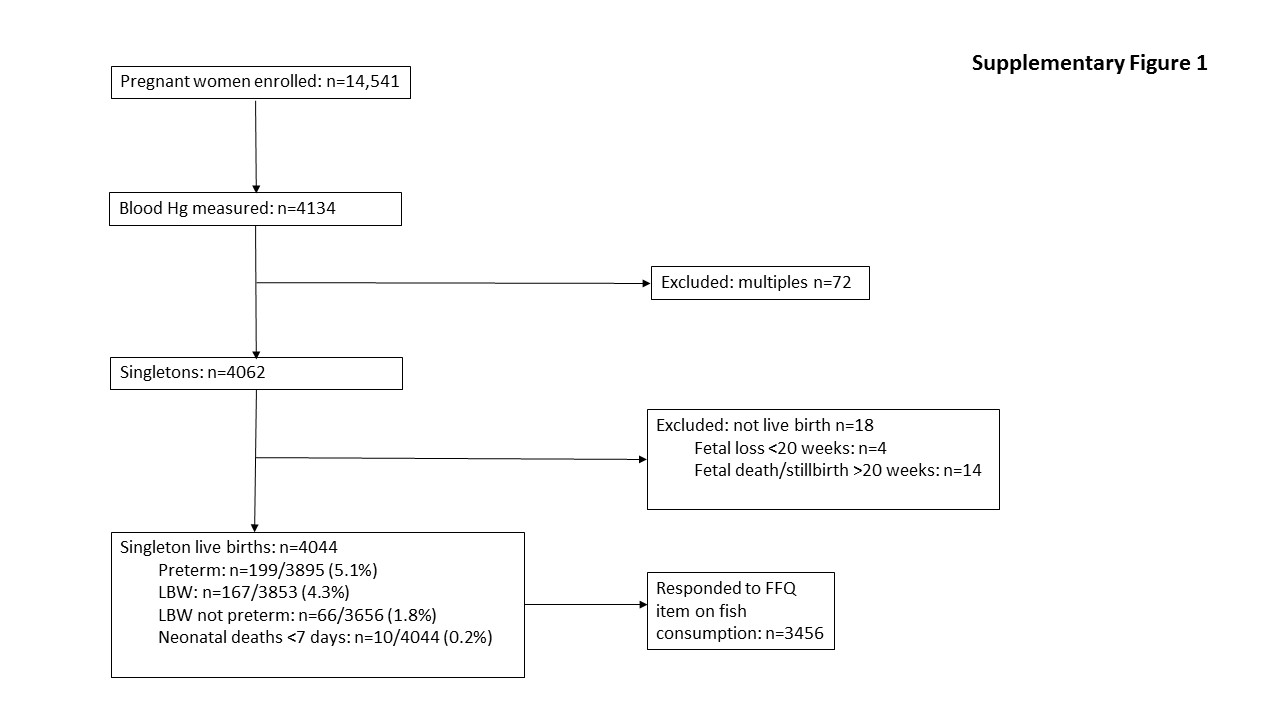

Supplement: Supplementary file 1 [file mmc1.docx]
